# Lectures on the Principles and Practice of Physic; Delivered at Kings College, London

**Published:** 1845-10

**Authors:** 


### BIBLIOGRAPHICAL NOTICES.

*Lectures on the Principles and Practice of Physic;* delivered at Kings College, London. By Thomas Watson, M. D. &c. &c. Second American from the Second London Edition, Revised, with Additions, by D. Francis Condie, M. D., Secretary of the College of Physicians; author of a Treatise on Diseases of Children, etc. etc. Royal 8vo.—pp 1060. Lea & Blanchard, Philadelphia, 1845.

This is confessedly one of the very best works on the principles and practice of physic in the English or any other language. From the press, both British and American, it has received the highest commendations, and, from the rapidity of its sale, we may infer that it has been equally well received by the profession at large. The original portion of the present publication being by these means so extensively known, and as extensively approved, it is only necessary for us at present to notice the contributions of the Editor, of the value of which our readers will probably anticipate our opinion, from his well known learning and experience.

On most of the subjects treated of by the author, his exposition of the views at present entertained by enlightened pathologists and therapeutists, is so full and satisfactory, as to have left little room for comment, and Dr. Condie has therefore judiciously confined himself mainly to the consideration of a few of the forms of disease more particularly interesting to the American physician, of which the account given by the author is somewhat defective, and one or two diseases endemic in the United States, and consequently omitted in the lectures by Dr. Watson. Some of these additional observations we shall now briefly notice.

Under the head of Aphthæ, Dr. Watson treats of several varieties of Stomatitis, as the simple *thrush,* or pultaceous inflammation of the mouths of young children, pseudo-membranous or diphtheritic inflammation, and follicular inflammation of the mouth, tongue and fauces, blending them together as one and the same disease. From this view Dr. Condie dissents. "The disease of the mouth occurring in infants during the period of suckling, in which the tongue, and the inner surface of the lips and cheeks, are covered, to a greater or less extent, with minute portions of a white matter resembling curd—and which constitutes the *muguet* of the French writers, the *thrush* or *children's sore mouth of nurses*, and the *aphthæ lactumina* and *aphthæ infantiles* of the older physicians, is evidently a very distinct affection from the *aphthæ* which occurs in the adult as well as in the infant. The first depends upon an erythematic inflammation of the mucous membrane of the mouth, in which an altered secretion, in the form of small and curd-like particles or flocculi, or, as in other diphtheritic inflammations, of large patches of a soft pseudo-membranous matter, takes place upon the surface of the inflamed membrane. Upon the separation of these morbid exudations, the membrane beneath is found to be smooth, and without any solution of continuity. According to Guersant, the curd-like exudation is deposited beneath the epithelium, and its separation is consequent upon the rupture of the latter; Plumbe is of a similar opinion; Guyot and Billard, however, never saw it, excepting upon the surface of the epithelium, and this accords with our own experience.

"We are to recollect that the disease just described (thrush) is the result of an erythematic inflammation of the mucous membrane of the mouth; *aphthæ*, however, are produced by a follicular inflammation of the membrane. This affection is more commonly observed about the period of dentition than at an earlier age—it is particularly liable to occur in children of a lymphatic temperament, or in whom hæmatosis has been rendered imperfect, by improper or innutritious food, a damp and cold, or impure and stagnant atmosphere, exclusion from the light, and neglect of cleanliness. It is, also, of very common occurrence during most of the chronic affections, especially of the intestinal canal, in persons of all ages."

In the propriety of this distinction, we coincide with Dr. Condie. Trousseau and Delpech maintain, that "the *muguet*, or *aphthous patches*, consist of a fibrinous pseudo-membrane, situated on the surface of the digestive mucous membranes, deprived [in those spots] of its epithelium,"—(Ranking's Half-yearly Abstract, Am.

Ed. p. 246)—which is little if at all more definite than the manner in which it is presented by Dr. Watson.

Under the head of *diarrhœa,* Dr. Condie has favoured us with a long note, containing views, both as to pathology and treatment, in accordance with those entertained by American practitioners generally, and consequently requiring no further comment.

On the subject of *dysentery,* the author is less full in his account than on most other topics, and Dr. Condie has therefore added largely to the text, especially in regard to the treatment. From his observations we should infer that his experience in the treatment of the disease must have been confined to sporadic cases, or at least such as were of a decidedly sthenic character—and we come to this conclusion both from the pathology he lays down, and the great reliance on blood-letting that he inculcates.

"In the ordinary cases of dysentery, he remarks, the morbid appearances detected after death are, inflammation with thickening of the mucous membrane of the colon and rectum; occasionally, mortification and sloughing of this membrane, but more generally, in protracted cases, deep and extensive ulcerations, in the course of the transverse bands of the colon, or enlargement and ulceration of the follicles of the large intestines." (p. 832,) This description refers to the dysentery of our own country, not the violent form witnessed in intertropical regions. That the cases described were more than ordinarily violent, maltreated, or protracted, is manifest from the post mortem appearances, as well as from the fact that they afforded opportunities for cadaveric examination at all; and it by no means follows from thence that the disease in its commencement is always severely inflammatory, or that the inflammation which may exist is unaccompanied by other lesions forbidding the loss of blood, as the following remarks imply. "There are few cases of dysentery in which the lancet, or the application of leeches or cups to the abdomen can be dispensed with, without endangering ulceration, thickening or other structural changes in the mucous membrane of the intestines—by which the sufferings of the patient are prolonged and his life endangered. . . . . . . The more violent forms of the disease can be successfully managed only by the prompt, free and even repeated use of the lancet, and the application, at the same time, of leeches upon that portion of the abdomen where pressure causes the most pain; and this system of active depletion must be continued, so long as fever or tenderness of the abdomen continues, more especially if the stools continue to be bloody."—(p. 834). Dr. Condie very properly condemns the *purging* treatment so unwarrantably pursued by some practitioners, and substitutes the revulsive and palliative effects of ipecacuanha, acetate of lead and opium, frequently combining all of those articles in his prescription. In regard to the use of the latter agent, we find the following remarks: "The intense sufferings which the patients often experience from the severe tormina and almost constant and distressing tenesmus attendant upon many cases of the disease, tempt the inexperienced practitioner to resort at once to opiates for their relief. But the use of opium in dysentery requires the utmost caution; until the violence of the inflammation, in the more severe forms of the disease is reduced by active depletion, they are, in general, inadmissible;" &c. After what he regards as sufficient depletion by leeches and the lancet, he resorts to moderate doses of opium to procure rest at night, or in combination with ipecacuanha, blue mass, &c. to relieve tormina.

It appears to us from the remarks we have quoted, and others for which we have not space, that our friend and townsman relies too much on bleeding and too little on the use of opium in this disease. We discover in his practice the remains of the doctrine that looked upon all febrile disturbances as evidences of inflammation, and all inflammations as necessarily demanding blood-letting. By the same class of therapeutists, opium has been regarded only as a stimulant, and all stimulants as inappropriate in the treatment of inflammation. In these views we do not concur. We have many instances of inflammation in which experience has shown that the abstraction of blood is inadmissible; cases too of no trifling importance; such as, for instance, strumous ophthalmia, inflammation of the throat in malignant scarlatina, dothinenteritis, pneumonia typhodes, some cases of erysipelas, &c. The inflammation which occurs in dysentery is in the mucous lining of the bowels, chiefly of the colon and rectum, and all experience, we believe, has shown that bleeding has comparatively little influence over inflammation in mucous surfaces—certainly much less than in the serous tissues. Dysentery, too, according to our observation, happens more frequently in adynamic conditions than those of a high sthenic character—as in hospitals, jails, camps, and endemico-epidemics. The dry and dark tongue, petechiæ &c. evince this, and consequently forbid active depletion in any form, and particularly by the abstraction of blood.

It is not to be understood by these remarks, that we discard bleeding in all cases of dysentery: on the contrary, we regard it in some instances, more especially in sporadic cases, as one of our most valuable remedies; but we think the precepts of Dr. Condie on this subject inculcate a too general and almost exclusive reliance upon it, and we have given our reasons for that belief. We think, also, that Dr. C. limits the use of opium too much. Whenever a patient is tortured with pain, no matter in what disease or under what circumstances, opium, in some of its forms, is proper; and if given in appropriate doses in the early stage of dysentery, with perfect rest, and appropriate diet, will not unfrequently cut short the disease; and in every stage of its progress, is demanded to relieve tormina and procure sleep, without which recovery is not to be expected.

On *typhous pneumonia* and *bilious remittent fever*, Dr. Condie has added extensive notes, characterized by much research and judicious observation; on the latter affection, particularly, his remarks constitute an excellent monograph, which may be consulted with advantage by even the best informed on the subject. From these, and other notes contained in this edition by Dr. Condie, we should be glad to make further extracts, as well as to make occasional comments, if we had not already exceeded the limits appropriated to this notice. As it is, we have only room left to say of this excellent work, that it is eminently deserving of the patronage of the profession, and that the value of the present edition of it is greatly enhanced by the additions of the editor.

